# *Drosophila *Uri, a PP1α binding protein, is essential for viability, maintenance of DNA integrity and normal transcriptional activity

**DOI:** 10.1186/1471-2199-9-36

**Published:** 2008-04-15

**Authors:** Jasmin Kirchner, Emese Vissi, Sascha Gross, Balazs Szoor, Andrey Rudenko, Luke Alphey, Helen White-Cooper

**Affiliations:** 1 Abbott Laboratories, Global Pharmaceutical Regulatory Affairs, Abbott Park, IL 60064-6157, USA; 2 Institute of Immunology and Infection Research, University of Edinburgh, EH9 3JT, UK; 3 Harvard University, FAS Molecular & Cell Biology, Sherman Fairchild Biochemistry Bldg, 7 Divinity Ave, Cambridge MA, 02138, USA; 4 Department of Zoology, University of Oxford, South Parks Rd, Oxford, OX1 3PS. UK

## Abstract

**Background:**

Protein phosphatase 1 (PP1) is involved in diverse cellular processes, and is targeted to substrates via interaction with many different protein binding partners. PP1 catalytic subunits (PP1c) fall into PP1α and PP1β subfamilies based on sequence analysis, however very few PP1c binding proteins have been demonstrated to discriminate between PP1α and PP1β.

**Results:**

URI (unconventional prefoldin RPB5 interactor) is a conserved molecular chaperone implicated in a variety of cellular processes, including the transcriptional response to nutrient signalling and maintenance of DNA integrity. We show that *Drosophila *Uri binds PP1α with much higher affinity than PP1β, and that this ability to discriminate between PP1c forms is conserved to humans. Most Uri is cytoplasmic, however we found some protein associated with active RNAPII on chromatin. We generated a *uri *loss of function allele, and show that *uri *is essential for viability in *Drosophila*. *uri *mutants have transcriptional defects, reduced cell viability and differentiation in the germline, and accumulate DNA damage in their nuclei.

**Conclusion:**

Uri is the first PP1α specific binding protein to be described in *Drosophila*. Uri protein plays a role in transcriptional regulation. Activity of *uri *is required to maintain DNA integrity and cell survival in normal development.

## Background

Biochemical analysis of protein phosphatase activity led to the identification of distinct enzyme classes based on sensitivity to inhibitors, *in vitro *substrate specificity and cation requirements. Type 1 protein phosphatase (PP1) is one of the major serine/threonine phosphatase classes found in all eukaryotic cells. Cloning of the catalytic subunits of PP1 (PP1c) revealed that there are distinct enzyme forms which had not been distinguished biochemically. Phylogenetic analysis has revealed that there is an evolutionarily conserved distinction between animal PP1α (human PP1α and γ; *Drosophila PP1α87B*, *PP1α13C *and *PP1α96A*) and PP1β (human PP1β or PP1δ; *Drosophila PP1β9C*) implying that the gene products have distinct biological functions despite their identical biochemical properties *in vitro *and >85% sequence identity [1]. In *Drosophila *larvae, PP1α87B provides 80% of the total PP1 catalytic activity [2], with 10% each being attributed to PP1α96A and PP1β9C [3]. PP1α87B and PP1β9C are both essential for viability, however PP1α96A and PP1α13C are dispensable [1,2,4,5].

PP1 has numerous diverse functions within the cell including regulation of cell cycle, cytoskeleton, transcription and synaptic plasticity [6-9]. While, *in vitro*, the catalytic subunit (PP1c) dephosphorylates a wide variety of substrates, the enzyme *in vivo *is found in a variety of complexes with different protein partners [10]. These PP1 interacting proteins target PP1c to specific subcellular locations, and modulate its activity towards different substrates. When complexed to a regulatory subunit, PP1c becomes much less promiscuous in its activity, so genuine PP1 interacting proteins tend to inhibit PP1c activity in standard *in vitro *assays (against phosphorylase *a *or myelin basic protein), even though their *in vivo *role is to promote PP1c's activity towards a specific substrate. At least 50 PP1c targeting subunits have now been described, including the G-subunit, that targets PP1c to glycogen particles, the M-subunit that targets PP1c to myosin, and Sara that targets PP1c to the TGFβ receptor [10-13].

The three human PP1c isozymes localise to different subcellular regions in both mitotically active cells and in terminally differentiated cells [14-17]. This suggests that there are targeting subunits that differentiate between the PP1c proteins. So far, mammalian neurabin I and neurabin II/Spinophilin, have been shown to selectively co-precipitate with mammalian PP1γ and PP1α in preference to PP1β [18,19]. Repo-man has a modest (3-fold) preference for PP1γ over PP1α, however the ability of Repo-man to distinguish between PP1γ and PP1β has not been reported [20]. Recent co-immunoprecipitation assays have identified a few more mammalian isozyme specific PP1-interacting proteins [21]. In *Drosophila*, one PP1β-specific binding protein has been described, MYPT-75D; this probably is important for mediating the single essential function of PP1β in flies, which is regulation of non-muscle myosin [3]. No *Drosophila *proteins with a preference for binding PP1α rather than PP1β have been described.

URI (unconventional prefoldin RPB5 interactor) has been implicated in modulation of the transcriptional response to nutritional cues in humans and *S. cerevisiae *[22]. URI mutant *S. cerevisiae *are viable, but constitutively over-express genes important for amino acid metabolism. *C. elegans uri-1 *mutant animals are also viable, but have defects in germ cell proliferation and DNA stability [23]. Human RMP (RPB5-mediating protein) is identical to URI, except that the clone described lacks the N-terminal 25aa. RMP was identified through its ability to bind the RPB5 subunit of RNA polymerase, and was demonstrated to have weak transcriptional co-repressor activity [24,25]. URI (lacking the first 75aa) has also been named NNX3 [26]. Here we present a functional analysis of the *Drosophila uri *gene.

## Results

### Uri is a PP1α specific binding protein

**Figure 1:**
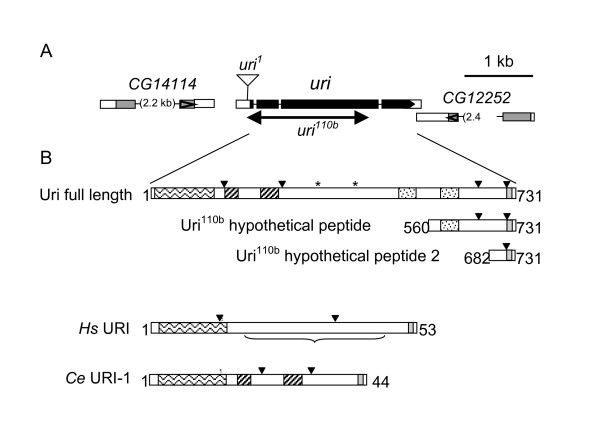
**uri genomic region, mutant alleles and protein domain architecture**. A. The *uri *genomic region adapted from FlyBase. The *uri *3' UTR overlaps with the 3' UTR of *CG12252*, encoded on the opposite strand. The position of the *P{GSV6}GS16344 *insertion in *uri*^1 ^is shown, as is the extent of the deletion in *uri*^110*b*^. B. Schematic diagram showing the similarity in domain architecture between *Drosophila *Uri, human URI and *C. elegans *URI-1 proteins. Wavy box – prefoldin domain; Striped box – coiled coil; Stippled box – repeat region; grey box – URI box (C-terminal conserved motif); asterisk – predicted PP1c binding site; inverted triangles – predicted NLS; bracket – region of human URI present in the RMP-D2 truncated protein. Truncated proteins that could be encoded by the transcript remaining in *uri*^110*b *^are indicated.

To identify potential regulatory subunits of the major protein phosphatase catalytic subunit of *Drosophila *we screened a yeast two-hybrid library using *Drosophila *PP1α87B as a bait and isolated 25 cDNAs representing 16 different genes. One of these genes (*CG11416*, *uri*) was represented by 2 independent clones. *CG11416 *has been described as the *Drosophila *homologue of URI since the N-terminal region (aa 30–124, wavy box in Figure 1B) contains a Prefoldin domain, most similar to that of human URI (RMP, NNX3), and the C-terminus contains a short region of homology termed the URI-Box (aa 720–729, grey box in Figure 1B) (Full alignment shown in [22], supplementary material). The *uri *predicted transcript encodes a protein of 731 amino acids with a calculated molecular weight of 84 kDa, although the protein runs at 110 kDa in SDS PAGE, and an isoelectric point (pI) of 4.66. Two coiled coil domains outside the prefoldin domain are predicted (striped boxes in Figure 1B). Human URI does not contain additional coiled-coil regions, but *C. elegans uri-1 *does. The overall acidity of the protein is partly explained by a very acidic region at aa 170–185. Human URI also has an acidic domain, as does worm *uri-1*. *Drosophila *Uri has three putative PP1c binding motifs ([KR]X{0,1} [VI]X [FW]) [27,28] at amino acids 337 (KVNF), 403 (RISF) and 469 (RNIEF), (asterisks in Figure 1B), while human URI and worm *uri-1 *each contain one PP1c binding motif (RVEF in human; KIKF in worm) at the end of the prefoldin domain. *Drosophila *Uri contains four predicted nuclear localisation signals (NLS) (inverted triangles in figure 1B); both human URI and worm *uri-1 *have two NLSs. The C-terminal region of *Drosophila *Uri contains a repeat sequence (from aa 504–568 and aa 587–651, stippled in Figure 1B) that shows no homology to the URI proteins from other species, or to any other protein sequence in the database (NCBI Blast).

**Figure 2:**
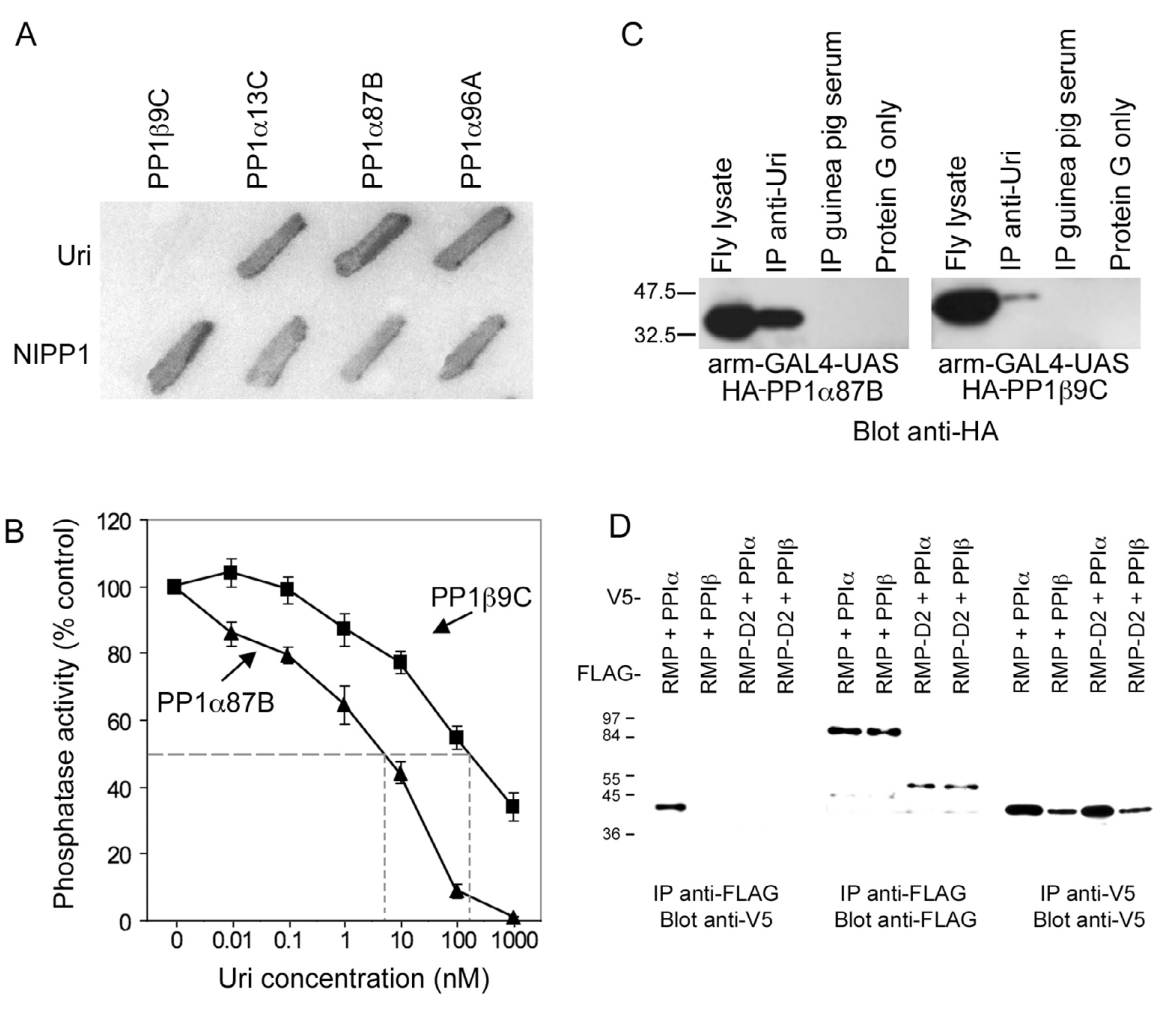
**Uri is a PP1α specific binding protein**. A. Uri binds all three *Drosophila *PP1α isozymes (PP1α13C, PP1α87B and PP1α96A) in yeast two-hybrid assays, but does not bind *Drosophila *PP1β (PP1β9C) in this assay. NIPP1 shows no discrimination between the isozymes, and is shown as a control. PP1 isoforms were expressed as DNA binding domain fusions (bait), while Uri and NIPP1 were Activation domain fusions (prey). B. Uri is an specific inhibitor of *Drosophila *PP1. The myelin basic protein phosphatase activity was measure in the presence of different concentrations of recombinant PP1α87B (triangles) or PP1β9C (squares). Phosphatase activity is shown as % of control in the absence of Uri. C. Uri immunoprecipitates ectopically expressed HA-PP1α87B more efficiently than HA-PP1β9C from fly extracts. Western blot showing similar levels of expression of HA-PP1α87B and HA-PP1β9C in total fly lysate, and proportion immuno-precipitated with the anti-Uri antibody. Normal guinea pig serum and Protein G sepharose only controls show no precipitation of the expressed proteins. D. Human PP1α (first lane) but not PP1β (second lane) co-immunoprecipitates with URI/RMP, and not URI/RMP-D2 (lanes 3 and 4) when co-expressed in COS7 cells (first panel). Expression controls are shown in the second and third panels.

The *Drosophila *PP1c genes encode proteins that are >85% identical to each other and have indistinguishable activities *in vitro*. Nevertheless, PP1β9C is structurally distinct from the PP1α isozymes and is encoded by an essential gene [1], suggesting some binding partners can distinguish between PP1c isozymes. Although *uri *was isolated in our small scale PP1α87B yeast two-hybrid screen, no *uri *clones were isolated in a 10-fold larger scale screen from the same library using PP1β9C as a bait [12], suggesting that Uri may be a PP1α-specific binding protein. We directly tested this in yeast two-hybrid, and found that Uri was able to bind all three PP1α forms, but was unable to interact with PP1β9C (Figure 2A).

PP1c dephosphorylates a wide range of substrates *in vitro*; substrate specificity *in vivo *is provided by targeting subunits. Addition of a PP1 targeting subunit will typically alter substrate specificity in the *in vitro *reaction, and therefore inhibit the ability of PP1c to dephosphorylate a wide variety of phosphosubstrates. We tested the ability of Uri to inhibit the myelin basic protein (MBP) phosphatase activities of recombinant PP1α87B and PP1β9C. Bacterially expressed Uri inhibited the PP1α87B MBP phosphatase activity with higher affinity than the PP1β9C MBP phosphatase activity. The Uri IC_50 _against PP1α87B was about 25-fold lower than that of Uri against PP1β9C (Figure 2B). No antibodies have been produced that can distinguish between the *Drosophila *PP1 proteins, so, to test the interaction between Uri and PP1α and PP1β *in vivo*, we ectopically expressed HA-tagged PP1α87B and PP1β9C proteins in flies, and tested their ability to co-immunoprecipitate with endogenous Uri. arm-GAL4 [29] flies have low-level ubiquitous expression of the yeast transcription factor Gal4p, which activates expression of transgenes under the control of the Gal4p target site, UAS. Immunoprecipitation of cell lysates of arm-GAL4; UAS-HA-PP1α87B and arm-GAL4; UAS-HA-PP1β9C flies with anti-Uri antibodies, followed by immunoblotting with anti-HA antibodies, showed that HA-PP1α87B co-precipitated more efficiently with Uri than did HA-PP1β9C (Figure 2C), consistent with PP1c activity assay data. The reciprocal experiment, immuno-precipitation with anti-HA antibodies and immunoblotting with anti-Uri antibodies confirmed this difference in the strength of the interactions (data not shown). Therefore Uri is the first *Drosophila *PP1c interacting protein to be shown to interact with PP1α with significantly higher affinity than with PP1β.

Since human URI also contains a predicted PP1c binding motif we investigated its ability bind different human PP1c isozymes. FLAG-tagged URI/RMP was transiently expressed in COS 7 tissue culture cells along with V5-tagged human PP1α or PP1β; PP1α was expressed more strongly in these experiments than PP1β. Immunoprecipitation of cell lysates with anti-FLAG antibodies followed by immunoblotting with anti-V5 antibodies showed that human PP1α co-precipitated efficiently with the human URI. Co-immunoprecipitation of PP1β and FLAG-tagged URI/RMP was not detected (Figure 2C). A deletion construct of human URI/RMP (RMP-D2) has been reported [25], the region of URI/RMP included in this construct is indicated by the bracket below the human protein in Figure 1. The putative PP1 binding sites in URI/RMP are missing in this deletion derivative. We found no co-immunoprecipitation between either of the PP1 isozymes and URI/RMP-D2. This consistent with the notion that the RVEF putative PP1 binding site on URI is important for the URI-PP1c association, although other sites missing in the truncated protein could also be implicated in the interaction. *Drosophila *Uri could also bind mammalian PP1α with higher affinity then PP1β when they were co-expressed in mammalian tissue culture (data not shown). Therefore, despite low overall sequence homology, the ability of Uri to discriminate between different PP1c isozymes is evolutionarily conserved.

### Uri protein is predominantly cytoplasmic

**Figure 3:**
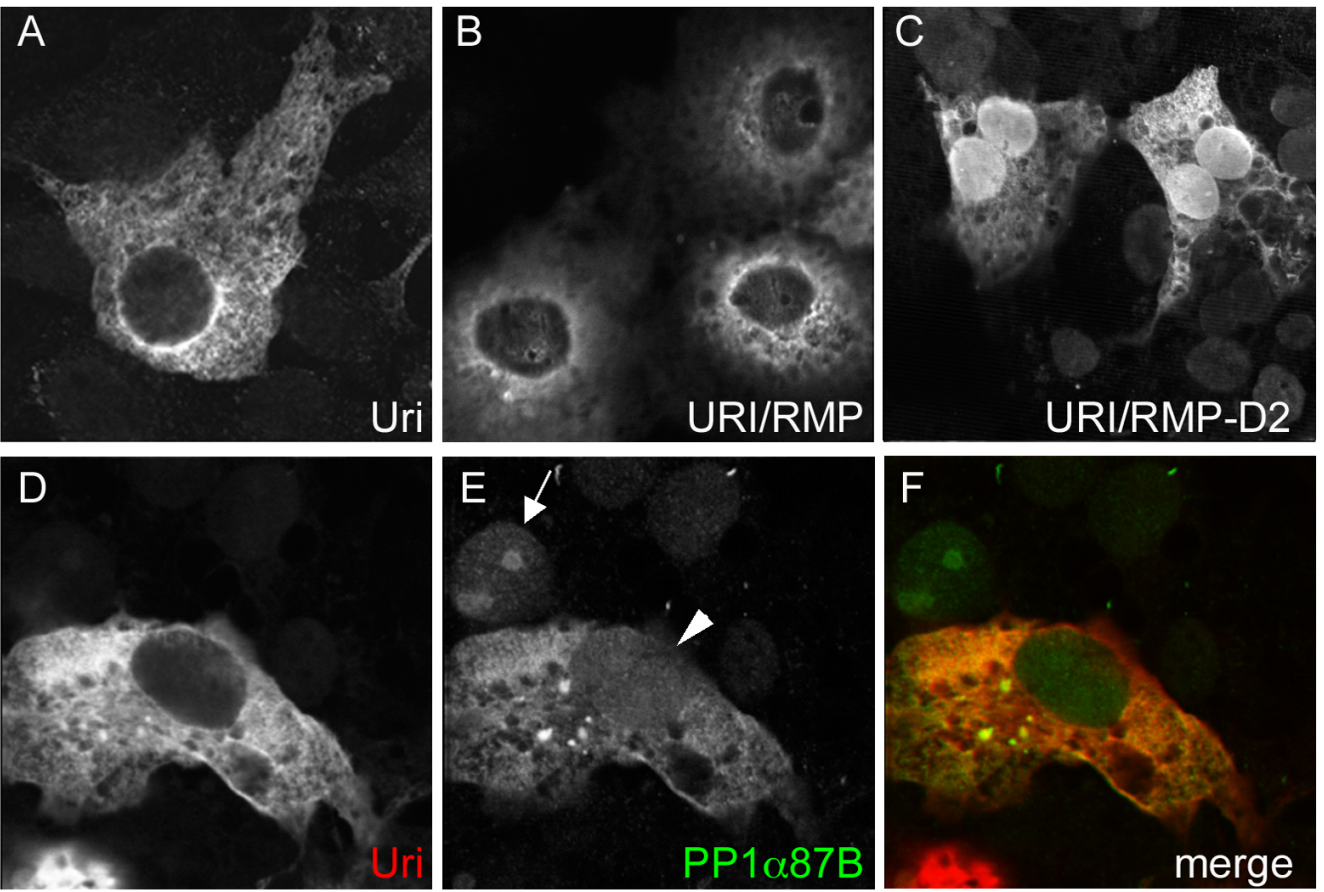
**Uri is cytoplasmic when expressed in COS7 cells, and can cause re-localisation of PP1α**. FLAG-tagged Uri (A), URI/RMP (B) or URI/RMP-D2 (C) were expressed in COS7 cells and visualised by immunofluorescence. Uri and URI/RMP were predominantly cytoplasmic, with a perinuclear concentration and weak nuclear staining. URI/RMP-D2 truncated protein predominantly localised to the nucleus, although cytoplamic labelling was also seen. D-F, COS7 cells transfected with FLAG-Uri (red) and HA-PP1α87B (green). The cell indicated by an arrow is only expressing HA-PP1α87B, and shows that HA-PP1α87B is nuclear when expressed in COS7 cells. The cell indicated by the arrowhead is expressing both HA-PP1α87B and FLAG-Uri, and shows a predominantly cytoplasmic co-localisation for Uri and HA-PP1α87B.

Human URI is an RPB5 binding protein [25]. This interaction, along with the predicted nuclear localisation sequence and the known role for human URI in transcriptional regulation, would predict that at least some URI protein should be nuclear and chromatin associated. To test this, we transiently expressed FLAG-tagged *Drosophila *Uri, human URI/RMP and URI/RMP-D2 and visualised their localisation by immunofluorescence. URI/RMP-D2 lacks the prefoldin domain but retains the ability to bind RPB5 in COS7 mammalian tissue culture cells [25]. We found that Uri (*Drosophila*) and URI/RMP (human) proteins were predominantly cytoplasmic with perinuclear localisation (Figure 3A, B). This is consistent with the cytoplasmic localisation for the N-terminally deleted NNX3 clone of URI/RMP [26]. When URI/RMP-D2 was expressed, strong nuclear localisation of the protein was observed (Figure 3C), indicating that the regions deleted from this construct are important for regulation of the nuclear localisation of URI protein. Human URI/RMP has been shown to bind DMAP1, a DNA methyltransferase-1 associated protein implicated in gene silencing, and this interaction promotes the nuclear re-localisation of URI/RMP [24]. To test whether the interaction between Uri and PP1 similarly altered the subcellular localisation of either protein, we co-expressed *Drosophila *Uri with PP1α87B in COS7 cells. PP1α87B, when expressed alone, can be detected in the cytoplasm, but is primarily nuclear (single transfected cell indicated by an arrow in Figure 3E). When PP1α87B was co-expressed with Uri, both proteins were more abundant in the cytoplasm; both the nuclear accumulation of PP1α87B and the perinuclear accumulation of Uri was lost (Figure 3D–F, arrowhead indicates nucleus of a co-transfected cell).

### *uri *is expressed throughout development, but is most abundant during embryogenesis, pupariation, and in adult gonads

**Figure 4:**
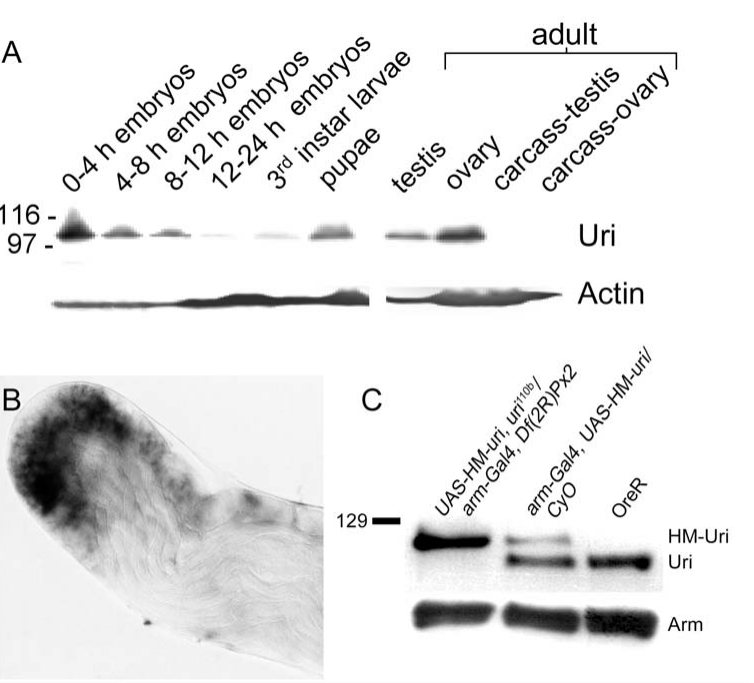
***uri *expression is highest in embryos, pupae and adult gonads**. A, Western blot showing Uri expression in extracts from various *Drosophila *developmental stages and tissues. Testis and ovary lanes contain gonads dissected from adults, carcass-testis is the male carcass after testes have been removed by dissection, similarly, carcass-ovary is female after ovaries have been removed. B, RNA *in situ *hybridisation to testis, the apical region of one testis is shown. *uri *expression extends from spermatogonia near the apical tip, to early primary spermatocytes, and declines in more mature cells further from the apical tip. C, Western blot showing endogenous Uri in wild type (OreR) wing disc extracts. HisMyc-Uri (HM-Uri) runs at a higher molecular weight, so two bands are present when this construct is expressed in a wild type background. Only the HM-Uri band was detected when HM-Uri was expressed in a *uri*^110*b*^/*Df(2R)Px2 *background, confirming that the mutant allele does not code for full length Uri protein. Arm protein is shown as a loading control.

We examined the developmental protein expression profile by Western blotting and found Uri protein to be most abundant in early embryos and pupae. The protein could not be detected in extracts of whole adult flies, or adults lacking gonads, however Uri was detected in extracts of ovaries and testes (Figure 4A). To determine the cellular and tissue distribution of *uri *transcription we used RNA *in situ *hybridisation. *uri *mRNA expression was uniform in embryos, imaginal discs, and larval brains (data not shown). In testes, *uri *was expressed in mitotically proliferating spermatogonia and in early primary spermatocytes, with staining levels decreasing as spermatocytes matured (Figure 4B). No transcripts were detected in post-meiotic stages. Male germline stem cells may express low levels of the mRNA.

### Uri protein is in cytoplasmic speckles *in vivo*

**Figure 5:**
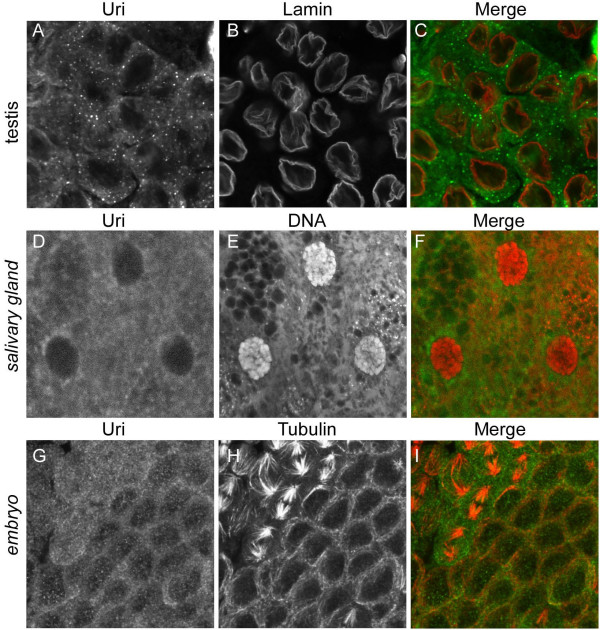
**Uri is predominantly cytoplasmic, and has a speckled distribution pattern in testes and embryos**. Immunofluorescence showing Uri localisation (A, green in C) to cytoplasmic speckles in wild type primary spermatocytes (A-C). The nuclear envelopes are labelled with anti-lamin antibodies (B, red in C). D-F, Salivary gland cells stained for Uri (D, green in merge) and DNA (E, red in merge) revealing that Uri is predominantly cytoplasmic, and is concentrated in the perinuclear region. G-I, Uri (G, green in I) is also localised to cytoplasmic speckles in interphase cells of cellularised early embryos. Cellular structure and mitotic regions were revealed by anti-tubulin staining (H, red in I). In cycle 14 mitotic domains (upper left region of figure) the Uri staining remained speckled, but was distributed uniformly in the cells.

Examination of protein subcellular localisations after ectopic expression can be complicated by artefacts associated with saturating the normal localisation machinery. Therefore we examined the subcellular distribution of endogenous *Drosophila *Uri, using the anti-Uri antibody, in tissues in which we know from Western blotting there are significant levels of Uri protein. In wild type primary spermatocytes (all stages), and maturing spermatids (not shown) Uri was found throughout the cytoplasm, with a distinctive concentration in small speckles (Figure 5A–C). No specific localisation to the nucleus was apparent. Persistence of Uri into post-meiotic stages indicates that it has a long half life, as no transcript was detected at this stage. To test whether the localisation in testis is simply an oddity of this tissue we examined Uri localisation in salivary glands and embryos. In salivary gland Uri was predominantly cytoplasmic, with a mild perinuclear accumulation (Figure 5D–F). In embryos we also found that Uri protein in interphase cells is primarily cytoplasmic, and some protein was in speckles in both the nucleus and the cytoplasm. The speckles and uniform staining persisted in mitotic cells, and no localisation to condensed chromosomes was found (Figure 5G–I).

### Uri protein is associated with transcriptionally active regions of polytene chromosomes

**Figure 6:**
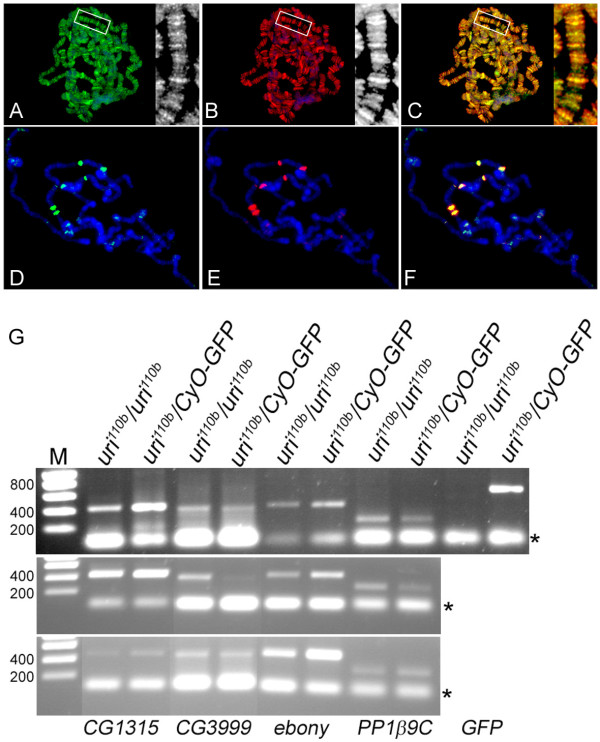
**Uri is on active chromatin in polytene chromosome spreads**. A-F, Polytene chromosomes from wild type larvae stained for Uri (green), active RNAPII (red) and DNA (blue). Higher power single channel greyscale images of the boxed region are shown in A, B, and the merge of these is shown in C. Uri co-localises with active RNAPII on normal larval polytene chromosomes (A-C; overlap is yellow in C.). D-F, After heat shock, RNAPII activity is restricted to the heat-shock puffs (E), Uri co-localises to these puffs (D, F). G, RT-PCR of potential *uri *target genes from *uri*^110*b *^homozygous embryos and *uri*^110*b*^/*CyO GFP *sibling control embryos. The results of three independent experiments are shown; size marker is shown on the left. mRNA levels of *CG3999 *and *PP1β9C *varied somewhat between experiments; *CG3999 *was slightly reduced in mutant compared to control embryos, *PP1β9C *on average was equal in mutant compared to control. *CG1315 *and *ebony *mRNA levels were significantly and reproducibly lower in the mutant embryos than in control embryos. GFP control primers confirm the accuracy of embryo selection based on fluorescence. Asterisks indicate primer dimer bands.

Although the majority of endogenous *Drosophila *Uri protein is cytoplasmic, the published interactions between Uri homologues and RNA polymerase II suggested that at least some Uri protein would be associated with chromatin. We examined the localisation of Uri on spreads of larval salivary gland polytene chromosomes and found that Uri stains numerous discrete bands. Only chromatin associated proteins are preserved for staining in these spread preparations, explaining the discrepancy between the chromatin localisation seen in spreads and the cytoplasmic localisation seen in whole mount. Co-labelling with an antibody recognising active RNA polymerase II revealed that the majority of the Uri positive bands are sites of active transcription (Figure 6A–C). *Drosophila *larvae, when stressed by heat shock, shut off most transcription and only actively transcribe from the heat shock response loci. This is associated with re-localisation of RNA polymerase II to a small number of heat-shock induced puffs on the polytene chromosomes [30]. We found that Uri also relocated to the heat shock puffs, and was lost from the remainder of the polytene chromosomes, on heat shock treatment (Figure 6D–F). Therefore, although most Uri protein is cytoplasmic in salivary gland cells, some is nuclear, and associated on chromatin with sites of active transcription.

### *uri *is essential for viability in *Drosophila*

Mutation of *C. elegans uri-1 *leads to sterility as well as multiple and variable somatic defects [23]. The budding yeast *URI *deletion strain is viable, but defective for expression of genes in the nutrient response pathway [22]. To determine the requirement for *uri *in *Drosophila *somatic and germ-line development we isolated loss of function mutant alleles. Searches of extant P-element insertion lines revealed that *P{GSV6}GS16344*, from the *Drosophila *Gene Search Project [31] was inserted 9 bp 5' of the annotated *uri *transcription start; we named this allele *uri*^1^. As the annotated 5' UTR was very short (28 bp), we used RT-PCR with primers upstream of this site to delimit the actual 5' end of the gene, and found that *uri *transcription started 112–208 bp upstream of the annotated transcription start. Therefore the *uri*^1 ^insertion is within the 5' UTR of *uri*, 37 bp upstream of the translation start site (Figure 1A).

The *uri*^1 ^chromosome was homozygous lethal, however *uri*^1^/*Df(2R)Px2 *males and females were viable, fertile, and did not exhibit a detectable phenotype (*Df(2R)Px2 *deletes the *uri *locus). Thus the lethality of the *uri*^1 ^chromosome was due to one or more second-site lethal mutations. *P*-element insertions in promoters or 5' UTRs often down-regulate transcription of the respective gene. Using RT-PCR designed against a region 3' of the *uri*^1 ^insertion, we found that there was considerably less *uri *transcript in *uri*^1^/*uri*^1 ^compared to *uri*^1^/*CyO, act-GFP *first instar larvae, showing that the *P *element in *uri *does indeed down-regulate *uri *transcription (data not shown). To generate stronger loss of function mutant alleles, we screened for deletions caused by imprecise excision of the *uri*^1 ^P-element and found nine that specifically disrupted *uri*. These deletions varied in size from 0.6 to 1.7 kb. We selected *uri*^110*b *^for further analysis as it has the largest deletion, removing the translation start, exon 2 and most of exon 3 (Figure 1A). The second site lethal mutation from the original chromosome was separated from the *uri*^110*b *^allele by meiotic recombination. Although *uri*^110*b *^deletes a significant proportion of the *uri *gene, we found that a transcript annealing most of the 5' UTR to the final 634 bp of the wild type transcript is expressed in *uri*^110*b *^mutant embryos. Two possible peptides encoded by this mutant transcript are shown (Figure 1B).

### *uri *is required for full expression of some genes in embryos

*uri*^110*b *^homozygote embryos hatch normally, as do *uri*^110*b*^/*Df(2R)Px2 *embryos. The mutant first instar larvae appeared sick, for example showing very little locomotion or feeding activity, and died soon after hatching. To determine whether this lethality could be attributed to defects in transcriptional activity we used RT-PCR to compare expression levels of several genes in mutant vs heterozygous sibling embryos. We chose to analyse expression of *ebony*, *CG3999 *and *CG1315*, the *Drosophila *homologues of *S. cerevisiae LYS2*, *GCV2 *and *ARG1 *respectively, which were shown to be regulated by *scUri *[22]. Expression of *ebony *and *CG1315 *was significantly and reproducibly lower in mutant embryos than wild type embryos, while expression of *CG3999 *was slightly elevated, or not altered in the mutant background (Figure 6G). Selection of a control gene in these experiments is not straightforward, standard choices such as a ribosomal protein are not necessarily appropriate when signalling downstream of TOR, which regulates growth and metabolism, could be affected. As a control we chose *PP1β9C*, which we had no reason to expect to change. Over several experiments expression of *PP1β9C *was somewhat variable between in mutant vs wild type, but there was never more than a 2-fold difference in expression of this gene in the two conditions. *Drosophila uri *is therefore essential for normal expression of at least two (probably more) target genes in embryos, but is not required for the expression of all genes.

### *uri *is required for cell viability in the germline

Expression of His-Myc tagged Uri was able to partially rescue the *uri*^110*b*^/*Df(2R)Px2 *lethality, so that flies of the genotype *UAS-HM-uri*, *uri*^110*b*^/*arm-GALl4, Df(2R)Px2 *were mostly late pupal lethal, with approximately 2% adult survivors. The surviving adults had weak locomotory ability and activity, and died within 24 hours. Nearly all of them exhibited additional wing vein material along the wing veins L2, L4, and L5; some pharate adults had small eyes. We found that expression of a hairpin *uri *RNAi construct in the eye imaginal disc gave a similar small eye phenotype (data not shown). We confirmed the absence of full length Uri protein in *uri*^110*b *^with Western blotting on wing disc samples from *UAS-HM-uri*, *uri*^110*b*^/*arm-GAL4, Df(2R)Px2 *third instar larvae (Figure 5C).

Uri protein is apparently gonad specific in adults (Figure 4A), we therefore wanted to examine the mutant phenotype in ovaries and testes. *UAS-HM-uri *is a *P{UAST} *derivative which does not express in the female germ-line [32], while *arm-GAL4 *does not express efficiently in the male germline. This lack of germline expression means that the *UAS-HM-uri*, *uri*^110*b*^/*arm-GAL4, Df(2R)Px2 *are essentially only rescued in the soma, allowing us to analyse the requirement for *uri *in the germline. These animals are developmentally delayed compared to wild-type, so their gonads had later stages of spermatogenesis or oogenesis than age matched controls.

**Figure 7:**
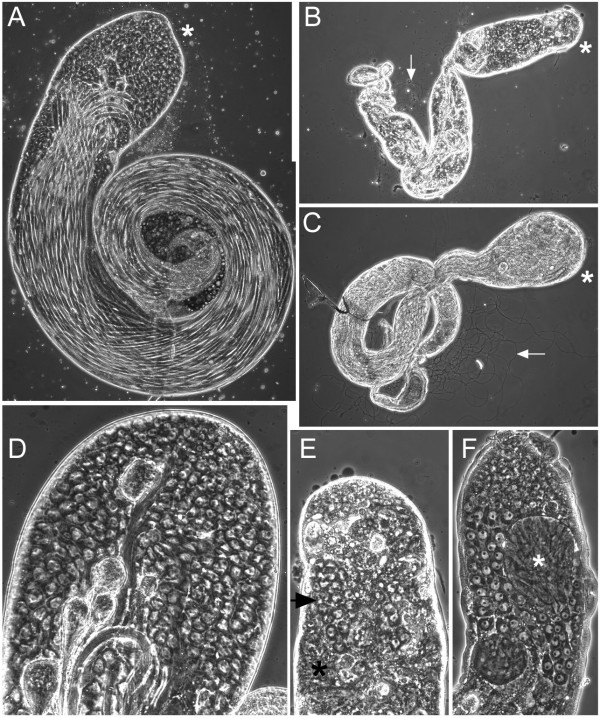
***uri *is required for male germline viability and differentiation**. A, wild type testis from male pharate adult. Stem cells reside at the apical tip (asterisk), cells move distally as they mature so most of the apical region is taken up by maturing primary spermatocytes. Elongating spermatids are seen pushing up the length of the testis. No motile sperm have yet developed. B, somatically rescued *uri*^110*b*^/*Df *testis from male pharate adult (UAS-HM-*uri*, *uri*^110*b*^/arm-GAL4, *Df(2R)Px2*), shown at the same scale as A. Some healthy spermatocytes near the apical tip (asterisk) and elongating stages are visible, as well as one motile sperm (arrow). Most of the testis is filled with degenerating dead cells. C, Testis from *uri*^110*b*^/*Df *male pharate adult rescued in the soma and germline from late spermatogonia (UAS-HM-*uri*, *uri*^110*b*^/arm-GAL4, *Df(2R)Px2*; Bam-GAL4-VP16/+), same magnification as A and B. The testis is significantly larger than without germline rescue, but smaller than wild type. Few early stage cells are seen (apical region marked with asterisk), but there are many differentiating spermatid bundles. Numerous motile sperm are visible (arrow). D, higher power view of apical region of wild type pharate adult testis. E, apical region of somatically rescued pharate adult *uri*^110*b*^/*Df *testis (genotype as B), same magnification as D. Healthy primary spermatocytes are indicated by the arrowhead, dead cells by the asterisk. F, apical region of *uri*^110*b*^/*Df *testis (genotype as C) from a pharate adult rescued in the soma and germline from late spermatogonia, same magnification as D and E. Fewer small cells than in wild type are present in the apical region, and post-meiotic spermatids (asterisk) are much closer to the apical tip.

Testes from *uri *somatically-rescued males were much smaller than their wild type counterparts (compare Figures 7A to 7B, and 7D to 7E). The testes contained a few apparently normal spermatogonia and spermatocytes (Figure 7E, arrow) as well as some elongated spermatids. Post-meiotic spermatids usually had abnormal morphology, although some testes contained a small number of normal motile sperm (Figure 7B, arrow). In addition to the healthy cells, mutant testes were full of dying cells and debris from dead cells (Figure 7E, asterisk). We were able to partially rescue the testis phenotype of somatically rescued *uri*^110*b*^/*Df(2R)Px2 *mutants by additional expression of *uri *in late spermatogonia and spermatocytes using the Bam-GAL4-VP16 driver [33]. These pharate adult males had longer testes than those lacking the germline expression (Figure 7C); the testes contained many later stages of spermiogenesis, and numerous motile sperm (arrow in Figure 7C). They were however not fully rescued, as they contained only a few cysts of spermatogonia and spermatocytes, and post-meiotic spermatids were located much closer to the apical tip of the testis than is normal (Figure 7F, asterisk, compare to 7D).

The ovarioles in ovaries of wild type pharate adult females typically have a germarium and three egg chambers, the oldest of which is at about stage 5 of oogenesis. Ovaries from *uri *somatically-rescued pharate adult females were small, and the germaria of these ovaries were thinner than wild type (data not shown). Most ovarioles lacked well defined stage 1 and later egg chambers, although one or two apparently normal later stage (up to stage 7) egg chambers were present in most ovaries.

### *uri *mutant cells contain damaged DNA

**Figure 8:**
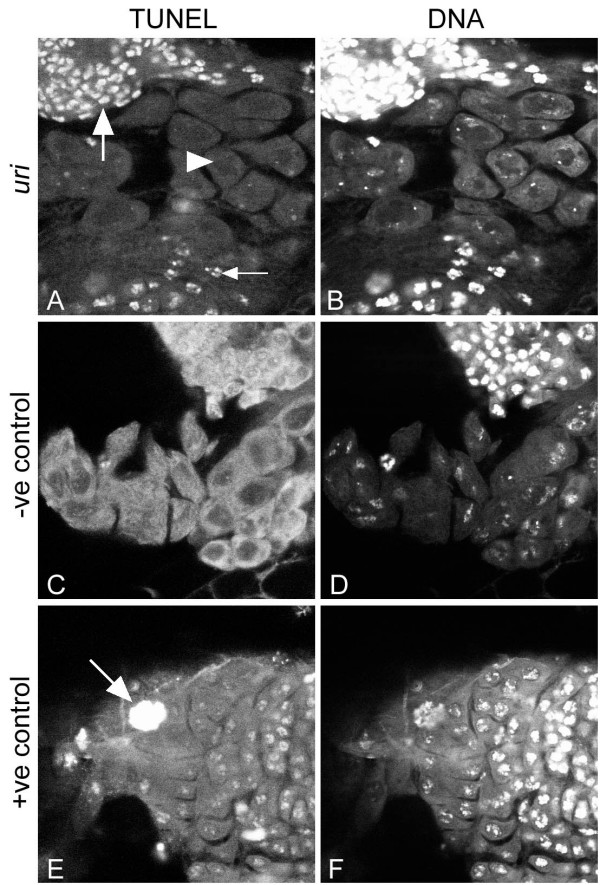
***uri *mutant cells accumulate DNA damage**. A, TUNEL labelling and B, DNA labelling, of a testis from a somatically-rescued *uri *mutant larva. Strong TUNEL staining was found on the DNA of spermatogonia (large arrow) and early primary spermatocytes (small arrow). Lower levels of TUNEL staining are seen in the oldest cysts present in these testes (mature primary spematocytes, arrowhead). C, D, wild type control larval testes show only background cytoplasmic TUNEL staining. E, F, TUNEL staining is seen on DNA of wild type larval testis cells after treatment with DNAse I (positive control). The large nucleus in this figure (arrow) is from the fat body.

*C. elegans uri-1 *mutants, while viable, were sterile due to germline proliferation failures caused by loss of DNA integrity. To test whether the *Drosophila uri *gene also has a role in DNA maintenance we examined testes of somatically rescued *uri*^110*b *^male larvae using TUNEL labelling. As a positive control we treated wild type testes samples with DNAse to induce DNA breaks, while untreated wild type testes served as a negative control. We observed high levels of TUNEL labelling of DNA in *uri *somatically rescued testes, indicating that these cells contained damaged DNA (Figure 8A, B). The most mature primary spermatocytes showed lower levels of TUNEL labelling. Negative control testes showed only background TUNEL labelling in the cytoplasm (Figure 8C, D). We also saw elevated levels of TUNEL labelling in somatic tissues, for example in fat body, from these larvae, consistent with their low viability to adulthood (data not shown).

## Discussion

*S. cerevisiae scUri (BUD27) *mutants have defects in bud site selection and in the transcriptional response downstream of TOR (target of rapamycin) signalling [22,34]. siRNA mediated depletion of *URI *also led to TOR response defects in human tissue culture cells [22]. *C. elegans uri-1 *mutants, while viable, had pleiotropic developmental defects and were sterile due to germline proliferation failures caused by loss of DNA integrity [23]. These apparently unconnected phenotypes suggest that *uri *is involved in multiple processes, making a strong case for its further characterisation.

Numerous lines of evidence implicate *uri *in transcriptional regulation. Both human and yeast URI proteins function as weak transcriptional repressors; *scUri *is also a context dependent activator [22,24,25]. Yeast URI (Bud27p) also binds to Gis1p's jmjC (histone demethylase) domain [35]. Human RMP (URI) was identified as a binding partner of RBP5, an RNA polymerase subunit. Consistent with this, we found that a fraction of endogenous Uri protein is associated with sites of active transcription on salivary polytene chromosomes and this association was maintained as the transcriptional profile changed in response to heat shock. This is consistent with a role for Uri in general transcriptional activation rather than repression.

Given the chromatin functions of Uri-containing complexes it is surprising that endogenous Uri is predominantly cytoplasmic. The URI/RMP prefoldin domain C-terminal half, with a predicted coiled-coil structure, acts as a cytoplasmic anchor in human cells [24]. This region interacts with DNA methyltransferase 1-associating protein, and this interaction promotes nuclear re-localisation. The putative PP1c interacting motif of the human protein also resides at the prefoldin domain C-terminus. Over-expression of *Drosophila *Uri prevented nuclear accumulation of co-expressed PP1α, indicating that PP1 does not promote Uri nuclear localisation (Fig 4). When ectopically expressed in human tissue culture cells, *Drosophila *Uri and human URI showed a perinuclear accumulation, this was also seen in salivary glands *in vivo*. In other cells, most obviously spermatocytes, Uri localised to cytoplasmic speckles. We are unsure what organelle or subcellular structures are associated with Uri speckles, although the speckles do not co-localise with the Golgi apparatus, or with P-bodies, which have similar speckled cytoplasmic localisation patterns in spermatocytes (data not shown).

Since both *ScUri *and human URI have been implicated in TOR signalling we examined expression in *uri *null embryos of *Drosophila *homologues of genes downstream of *scUri*. Two of the genes tested, *CG1315 *and *ebony *were reproducibly expressed at lower levels in mutant embryos. Thus, in contrast to the yeast situation, *uri *is required for full expression of these genes. TOR signalling in *Drosophila *is important for larval growth; mutant larvae grow slowly, but live for up to 30 days [36]. *uri*^110*b *^larvae die soon after hatching and are not developmentally delayed, indicating that, although *uri *may be implicated in regulation of TOR target genes, it probably has a wider range of target genes and/or other cellular functions. Further examination of the transcript profiles in mutant animals, for example by microarray analysis, would reveal the full extent of the transcriptional defect in *uri *mutant larvae. Human URI was isolated in a complex that also contained TIP48, TIP49, RBP5 and several small prefoldin domain proteins [22]. Like *uri*, *Drosophila *TIP48 (reptin) and TIP49 (pontin) mutant die as first instar larvae with no obvious defects [37], so *uri *lethality could potentially be attributable to defects in a complex containing these ATPases.

Uri protein is most abundant in embryos, pupae and in adult gonads; where expression is higher in germline than soma. This germline enrichment of fly Uri correlated well with the sterility phenotype in worms, and led us to investigate *uri*'s role in gonads in more detail. Partial somatic rescue of *uri *mutant flies enabled us to analyse the cell autonomous germline role, and revealed strong effects in both males and females. In both sexes the major defect was reduced cell viability. Spermatogenesis in flies is maintained by a population of stem cells, which give rise to spermatogonia. Defects in stem cell self renewal or survival eventually lead to empty (or nearly empty) testes, as stem cells are not maintained. Similarly, defects in spermatogonial survival lead to extremely small testes, as only stem cells remain. The somatically rescued *uri *male phenotype is consistent with loss of stem or early spermatogonial cells. The testes were mostly filled with dead or dying cells, indicating that *uri *is required for cell viability. Very few late spermatids were found, although we could occasionally see motile sperm. These would have initiated spermatogenesis in early larvae, and may have been saved by perdurance of maternally provided Uri protein. Provision of Uri to the germline using Bam-Gal4-VP16, which expresses in late spermatogonia and early spermatocytes, partially rescued the testis phenotype. Many more later stages of spermatogenesis were seen, indicating that *uri *is required for the viability of late spermatogonia and spermatocytes. Testes rescued by expression of *uri *with Bam-Gal4-VP16 had fewer early spermatogonia than wild type testes. The inefficient rescue of these cells show that *uri *is required in all spermatogonia, and possibly also in germline stem cells, to maintain cell viability. The RNA *in situ *confirms that *uri *is most highly expressed in spermatogonia and early spermatocytes. Somatically rescued *uri *females had thinner germaria than wild type, and mostly lacked later oogenesis stages.

*C. elegans uri-1 *is important to maintain DNA stability in the worm germline. By analogy with worms, the cell death could be due to accumulation of DNA damage; it could also be due to transcriptional defects, as *uri *is required for transcription. We established that *uri *mutants have defects in maintenance of DNA integrity, as shown by TUNEL staining. This is in complete agreement with the *C. elegans *findings. However, we cannot rule out the possibility that primary defects in transcriptional regulation lead to the DNA damage phenotype as a secondary effect.

Most PP1c interacting proteins do not discriminate between PP1α and PP1β isozymes. MYPT-75D was the first *Drosophila *protein to show differential binding, having higher affinity to PP1β than PP1α. This specificity is linked to the essential role of PP1β in flies – non-muscle myosin regulation [3]. Uri is the first *Drosophila *protein to be demonstrated to have a strong preference for PP1α over PP1β. An essential, non-redundant function for PP1α is suggested by inability of PP1β to rescue PP1α mutants [5]. Lethality of *uri *mutants supports the notion that PP1α has a role that cannot be supplied by PP1β. Uri can bind all the *Drosophila *PP1α isozymes, and indeed was identified as a PP1α96A putative interacting protein in a large scale yeast two-hybrid screen [38]. Canonical PP1c binding sites in human and worm URI suggested that binding to PP1 was probably conserved for this protein. We confirmed the human URI-PP1c interaction directly, adding URI to the ever-growing list of PP1c binding proteins. More excitingly, we show that interaction with PP1α in preference to PP1β is conserved between fly and mammalian URI.

## Conclusion

Here we have shown that *Drosophila uri*, is an essential PP1α-specific binding protein. Using genetic and biochemical analyses we implicate *uri *in regulation of transcription, germ-line and somatic cell viability and maintenance of DNA integrity.

## Methods

### *Drosophila *culture and strains

Drosophila were cultured on standard yeast/glucose/maize (or wheat flour) media, at 25°C. Wild-type was OregonR. *P{GSV6}GS16344 *was generated by DGSP [31] and kindly provided by Toshiro Aigaki (Tokyo Metropolitan University, Japan). Bam-Gal4-VP16 was provided by Dennis McKearin [33]. Other lines used were obtained from Bloomington *Drosophila *stock centre, and are described in Flybase [39]. *UAS-HM-uri *transformants were selected by standard techniques after injection of pP{UAS-HM-*uri*} into *w*^1118 ^embryos. The *uri*^110*b *^deletion was made by selection of *w*^- ^excisions of the *P{GSV6}GS16344 *element using CyO, Δ2–3 as a transposase source. In total 509 excision lines were generated, of which 31 were lethal in trans to *Df(2R)Px2*. These were tested by PCR to identify which genes in the region were affected (*CG11414*, *uri *or *CG12252*).

### Yeast two-hybrid screen

A two-hybrid *Drosophila *third instar larval cDNA library constructed in pACT [40] was screened in the yeast strain Y190 using a full length PP1α87B cDNA fused to GAL4 (pAS2-PP1α87B) as a "bait", as described in [41]. Two independent clones of *uri *(CG11416) were isolated.

### Molecular cloning and plasmid construction

A full-length *uri *cDNA was created from the longest partial EST available at the time (LD39507) whose 5' end is 13 bp downstream of the *uri *ATG initiation start codon. The cDNA was amplified by PCR using Platinum *Pfx *DNA Polymerase (GIBCO BRL) with the missing sequence, to the ATG incorporated within the 5' primer. This yielded a 2437 bp PCR product that was directionally cloned into *NdeI/NotI *sites of pGBKT7 for yeast two-hybrid screening. This *uri *fragment was subcloned into FLAG-pcDEF3 for mammalian tissue culture expression, into pET-28m for bacterial expression, and into pP{UAS-HM} [42] to create pP{UAS-HM-*uri*} for *Drosophila *transgenesis.

The pAS2-PP1c constructs used contain full length PP1c (PP1α87B, PP1α96A, PP1α13C or PP1β9C) fused to the DNA binding domain of GAL4 [41]. A partial cDNA clone of the *Drosophila *homologue of NIPP1 (NIPP1Dm) fused to the activation domain of GAL4 in pACT served as a positive control for two-hybrid interactions. pNKFlag-RMP and pNKFlag-RMP-D2 mammalian expression constructs were kindly provided by Seishi Murakami (Kanazawa University, Japan). Mammalian tissue culture expression constructs of human PP1α (clone H-X70848M) and human PP1β (clone H-X80910M) in the vector pcDNA3.1/GS were from Invitrogen. The *Rpb5 *ORF was amplified by PCR from *Drosophila *genomic DNA and subcloned into the pET28a bacterial expression vector (Novagen).

### Preparation of protein extracts from flies

Adult females were collected and either stored at -80°C or used immediately. Flies were homogenized at 4°C in IP buffer (25 mM HEPES, pH 7.5, 100 mM NaCl, 1 mM EGTA, 0.1% Triton X-100, 10% glycerol containing EDTA-free protease inhibitor mix (Roche, Indianapolis)). Homogenates were clarified by centrifugation (20 min at 10000 × g, 4°C) and the supernatants used in immunoprecipitation and pulldown experiments.

### Western Blotting

For developmental western blotting, embryos, larvae, pupae and flies were collected, frozen, homogenised in 2 × SDS sample buffer and boiled for 10 min. Samples were stored at -20°C and spun again before loading. For wing disc samples, 20 wing imaginal discs were taken up in 10 mM Tris-HCl pH 6.8, 180 mM KCl, 50 mM NaF, 1 mM NaVO_4_, 10 mM β-glycerolphosphate, 1% Triton X-100, 0.1% Tween 20 and stored at -80°C. An equal volume of 2 × SDS sample buffer was added before boiling and loading. Protein extracts were run on 10% SDS polyacrylamide gels, and transferred to Immobilon-P PVDF nylon membrane. Western blots were stained with Ponceau S, washed and blocked with 5% non-fat dried milk and then probed sequentially with the primary and HRP conjugated secondary antibodies (Sigma). Detection was by Supersignal chemi-luminescence (Pierce).

### Preparation of recombinant proteins and phosphatase assays

Recombinant NH_2_-terminal His_6_-tagged PP1β9C, PP1α87B, (cloned into pET28a) and Uri (pET28m) were expressed in *E. coli *BLR21 (DE3) cells. His_6_-tagged proteins were purified using NiNTA agarose (Qiagen) following the manufacturer's instructions. Renaturation of PP1c was carried out as described in [43]. Recombinant PP1β9C was expressed in *P. pastoris *as described in [44] and was used for the myelin basic protein phosphatase (MYBPP) assays. The myelin basic protein phosphatase (MYBPP) assays were performed using a Protein Serine/Threonine Phosphatase (PSP) Assay System (New England Biolab) and [^32^P] ATP (5000 Ci/mmole from Amersham Pharmacia Biotech). One unit of MYBP phosphatase is defined as the amount of enzyme which releases 1 nanomole of [^32^P] phosphate/minute from ^32^P labelled MYBP in the standard assay.

Transient transfection of COS7 mammalian cells was achieved using FuGENE 6 transfection reagent (Boehringer Mannheim) according to the manufacturer's instructions. Recombinant proteins were detected by indirect immunofluorescence and confocal microscopy.

### Antibodies

Anti-Uri polyclonal antibodies were generated by immunising guinea pigs with recombinant full length bacterially-expressed Uri protein (Moravian-Biotechnology). Guinea pig anti-Uri antibody was used at a dilution 1:500–1000 for Western blotting and 1:100 for immunofluorescence (1:20 for polytene chromosome). Anti-lamin antibody (T47, monoclonal supernatant) was kindly provided by D. Glover (Cambridge University, UK) and was used at 1:20–50 dilution for immunofluorescence. Anti-RMP antibody was used for Western blotting at 1:2000 dilution and was kindly provided by Seishi Murakami (Kanazawa University, Japan). Anti-RNAPII H14 (Covance/BabCo) was used at 1:500 dilution for Western blotting and at 1:100 for immunofluorescence. Anti-V5 antibody (Invitrogen) was used at a dilution 1:1000 for Western blotting. Anti-Arm was used at 1:300 (DSHB, Iowa). Secondary antibodies coupled to Cy3, Cy5, FITC and Alexa 488 were used at a dilution 1:1000 (Jackson or Molecular Probes) for immunofluorecence. Secondary antibodies coupled to HRP were diluted 1:10000 for Western blotting.

### Immunoprecipitation from flies

Lysates were pre-cleared by addition of 50 μl of Protein G Sepharose resin (Pharmacia). Following a 15 minute incubation on ice this mixture was centrifuged at 4°C for 1 minute 2500 × g. Aliquots of cleared lysates with 250 μg total protein content were withdrawn and used in IP experiments. After incubation of cleared lysates with the antibody required, 50 μl of equilibrated Protein G Sepharose was added and incubated for 1 hour on ice. Then resin was pelleted as before and washed 3 times with ice cold IP buffer. The beads were finally separated by centrifugation at 4°C 20 min at 10 000 × g, resuspended in 2 × SDS sample buffer and subjected to western blotting.

### Immunoprecipitation from COS7 cells

Transfected COS7 cells were washed twice in PBS and lysed in buffer containing 50 mM Tris-HCl pH (7.4–8.0), 0.5% Triton-X100, 150 mM NaCl, protease inhibitors (Roche). After centrifugation, supernatants were incubated with 1–2 μg antibody for 1–3 h on ice with gentle agitation. Equilibrated protein G-Sepharose was added and incubated for 1 hour, pelleted and washed with lysis buffer. Beads were resuspended in 2 × SDS-PAGE sample buffer and subjected to Western blotting.

### Immunofluorescence and *in situ *hybridisation

For tissue culture immunofluorescence, mammalian COS7 cells were grown on coverslips, transfected as described above, fixed with 4% paraformaldehyde for 10 minutes and permeabilized with methanol. Samples were blocked in 10% FCS in PBS for 1 hr and then were incubated overnight at 4°C with the primary antibody in blocking solution. After washes the coverslips were incubated with fluorescence labelled secondary antibody for 2 hours at room temperature, were mounted on slides, and examined by confocal microscopy. Immunofluorescence staining of *Drosophila *embryos and intact salivary glands after formaldehyde fixation, and methanol devitellinisation for embryos, was carried out using standard protocols; testes were stained as in [45]. For *in situ *hybridisation an anti-sense *uri *dig-labelled RNA probe was made by in vitro transcription using partial cDNA in pBluescript KS as a template, and Roche Dig-RNA labelling mix according to manufacturer's instructions. The probe was hydrolysed to give an average length of 100 nucleotides, hybridisation was carried out as described [46].

### Staining of larval salivary gland polytene chromosomes

Immunostaining of polytene chromosomes was performed as described by [47], with minor modifications [48]. For heat shock experiments, larvae were heat shocked at 37°C for 20 min in a water bath and salivary glands were dissected in PBS warmed to 37°C to prevent recovery. DNA was detected with Hoechst 33258 (0.5 μg/ml in water). Slides were mounted in 85% glycerol/PBS/2.5% n-propyl-gallate. Images were collected using a Q-imaging Retiga 1300 digital camera mounted on an Olympus BX50 epi-fluorescence microscope.

### RNA extraction and RT-PCR

For reverse transcription PCR (RT-PCR) non-fluorescent *uri*^110*b *^(or *uri*^1^) homozygotes were sorted from their fluorescent *uri*^110*b *^(or *uri*^1^)/*CyO, act-GFP *heterozygous siblings. For semi-quantitative RT-PCR on *uri*^110*b *^three (18.5–20.5 hours after egg laying) embryos were pooled, and total RNA was extracted using Trizol (Invitrogen). cDNA was generated from all the extracted RNA using Superscript II (Invitrogen), and 1/40^th ^of the cDNA reaction product was used as a template for each PCR (30 amplification cycles for *ebony*, 35 cycles for *CG1315*, *CG3999*, *PP1β9C *and *GFP*). To analyse expression of *uri *transcripts, and to map the 5' end of *uri*, RNA was extracted from first instar larvae and processed as above.

### TUNEL labelling of testes

TUNEL labelling was carried out with the "In situ cell death detection, Fluorscein" kit (Roche). Testes were dissected from male larvae in testis buffer, transferred to a 20 μl drop of testis buffer on a poly-l-lysine treated slide, cut open, and 40 μl of 4% paraformaldehyde in PBS was added. After 15 minutes the cells were squashed by addition of a coverslip which was then flipped off after freezing in liquid nitrogen. Slides were washed briefly in PBS, permeabilised in PBS+ 1% Triton + 0.5% acetic acid, rinsed in PBS then permeabilised again in 0.1% Triton, 0.1% (Tri-)Na Citrate for 4 min. As a positive control one slide was treated with DNAse for 10 minutes. Labelling was carried out for 1 hr at 37°C in a humid chamber using 5 μl enzyme solution and 45 μl label solution per slide. After washing with PBS the slides were incubated with 1 mg/ml RNase A for 15 min or 0.5 mg/ml RNase A overnight at 4°C, counter-stained with propidium iodide (1 μg/ml in PBS), mounted and observed with confocal microscopy.

## Authors' contributions

JK made the *uri *mutant and did the phenotypic analysis. EV made the anti-Uri antibody and did immunostainings, immunoprecipitation and Western blotting. SG identified Uri as a PP1α specific binding protein. BS did the protein phosphatase assays. AR did immunostaining of polytene chromosomes. HW-C supervised aspects of the work, assisted with immunostainings, *in situ *hybridization and phenotypic analysis, and wrote the paper. LA cloned *uri *in the original yeast 2-hybrid screen did *in situ *hybridisation, and supervised aspects of the work. All authors read and approved the final version of the manuscript.
